# Orphanhood and Caregiver Loss Among Children Based on New Global Excess COVID-19 Death Estimates

**DOI:** 10.1001/jamapediatrics.2022.3157

**Published:** 2022-09-06

**Authors:** Susan Hillis, Joel-Pascal Ntwali N’konzi, William Msemburi, Lucie Cluver, Andrés Villaveces, Seth Flaxman, H. Juliette T. Unwin

**Affiliations:** 1 Global Reference Group on Children Affected by COVID-19, University of Oxford, Oxford, United Kingdom; 2 African Institute for Mathematical Sciences, Kigali, Rwanda; 3 Division of Data, Analytics, and Delivery for Impact, World Health Organization, Geneva, Switzerland; 4 Department of Social Policy and Intervention, Oxford, and Department of Psychiatry and Mental Health, University of Cape Town, Cape Town, South Africa; 5 CDC COVID-19 Response Team, US Centers for Disease Control and Prevention, Atlanta, Georgia; 6 Department of Computer Science, University of Oxford, Oxford, United Kingdom; 7 MRC Centre for Global Infectious Disease Analysis and the Abdul Latif Jameel Institute for Disease and Emergency Analytics (J-IDEA), School of Public Health, Imperial College London, London, United Kingdom

## Abstract

This study assesses estimates of new orphanhood based on excess deaths to provide a comprehensive measure of the COVID-19 pandemic’s long-term impact on orphanhood and caregiver loss.

The availability of new excess mortality data enables us to update global minimum estimates of COVID-19 orphanhood and caregiver death among children.^1,2,3,4^ Consequences for children can be devastating, including institutionalization, abuse, traumatic grief, mental health problems, adolescent pregnancy, poor educational outcomes, and chronic and infectious diseases.^4,5^ Global totals and country comparisons were previously hampered by inconsistencies in COVID-19 testing and incomplete death reporting. The new orphanhood estimates derived here based on excess deaths provide a comprehensive measure of COVID-19’s long-term impact on orphanhood and caregiver loss.

## Methods

Using previous methodology for combining age-specific death and fertility rates,^4^ we use Guidelines for Accurate and Transparent Health Estimates Reporting (GATHER) reporting guideline for this epidemiologic modeling study to update COVID-19 estimates of parent and caregiver loss. We computed excess mortality-derived estimates for bereft children in every country, using data from the World Health Organization (WHO), *The Economist*, and the Institute for Health Metrics and Evaluation (IHME).^1,2,3,6^ We replaced COVID-19 deaths in previous logistic models with excess deaths (except when excess deaths were negative) to generate composite deaths for January 1, 2020, through December 31, 2021, and for January 1, 2020, through May 1, 2022 (eMethods in the Supplement; Table). We used bootstrapping to calculate uncertainty around estimates from fertility and death data. We present regional and national estimates using WHO-based mortality methods.

**Table.** Estimates of Orphanhood and Caregiver Loss Using Adjusted Excess Deaths^a^

Data source	No. (95% credible interval)			
	Composite deaths	Orphanhood	Orphanhood and primary caregiver loss	Orphanhood and primary and/or secondary caregiver loss
**December 31, 2021**				
*The Economist*	18.0 (14.4-21.5)	9.2 (7.5-10.9)	9.7 (8.0-11.5)	12.3 (9.8-14.8)
IHME	18.3 (17.7-18.8)	9.7 (9.1-10.3)	10.3 (9.7-10.9)	12.9 (12.2-13.5)
WHO	15.6 (13.9-17.3)	6.9 (5.8- 8.0)	7.2 (6.1-8.4)	9.5 (8.1-11.0)
**May 1, 2022**				
*The Economist*	21.3 (17.2-25.4)	11.0 (9.1-12.9)	11.6 (9.7-13.6)	14.8 (11.9- 17.6)
IHME	20.5 (19.9-21.1)	10.6 (10.0-11.2)	11.2 (10.6-11.9)	14.1 (13.4-14.8)
WHO	17.5 (15.7-19.3)	7.5 (6.4-8.7)	7.9 (6.7-9.2)	10.5 (8.9-12.0)

Abbreviations: IHME, Institute for Health Metrics and Evaluation; WHO, World Health Organization.

^a^ Estimates are reported in millions and used *The Economist*, IHME, and WHO excess data through 2021 (end of reporting period for IHME and WHO data sets) and adjusted using Johns Hopkins University data through May 1, 2022. Composite death data are calculated as the maximum COVID-19 and excess deaths at the country level. We used excess deaths for most countries because excess deaths tend to be greater than reported COVID-19 deaths; in those countries where excess deaths are negative or lower than COVID-19 deaths (for example, because of lockdown-associated reductions in motor vehicle fatalities and other types of injuries), we derived pandemic orphanhood and caregiver loss estimates from reported COVID-19 deaths.

## Results

Using WHO excess mortality (more conservative than findings from IHME and *The Economist*), we estimate that 10 500 000 children lost parents or caregivers (Table), and 7 500 000 children experienced COVID-19–associated orphanhood through May 1, 2022. Greater numbers affected by orphanhood by primary and/or secondary caregiver loss were found in the Africa (24.3% [95% credible interval [CI], 19.3%-27.6%]) and Southeast Asia (40.6% [95% CI, 35.3%-46.2%]) WHO regions, compared with the Americas (14.0% [95% CI, 12.6%-15.8%]), Eastern Mediterranean (14.6% [95% CI, 12.9%-16.2%]), European (4.7% [95% CI, 4.4%-5.3%]) and Western Pacific (1.8% [95% CI, 1.7%-1.9%]) regions through May 1, 2022 (Figure A). Similarly, variation in estimates arises at national levels, with India (3 490 000 [95% CI, 2 430 000-4 730 000]), Indonesia (660 000 [95% CI, 390 000-1 020 000]), Egypt (450 000 [95% CI, 360 000-540 000]), Nigeria (430 000 [95% CI, 40 000-900 000]), and Pakistan (410 000 [95% CI, 80 000-770 000]) worst affected through May 1, 2022 (Figure B). Among the WHO regions most affected, countries with the highest numbers of bereaved children in Southeast Asia included Bangladesh, India, Indonesia, Myanmar, and Nepal and in Africa included Democratic Republic of Congo, Ethiopia, Kenya, Nigeria, and South Africa. Our updated Orphanhood Calculator^6^ provides these new numbers for every country.

**Figure.**
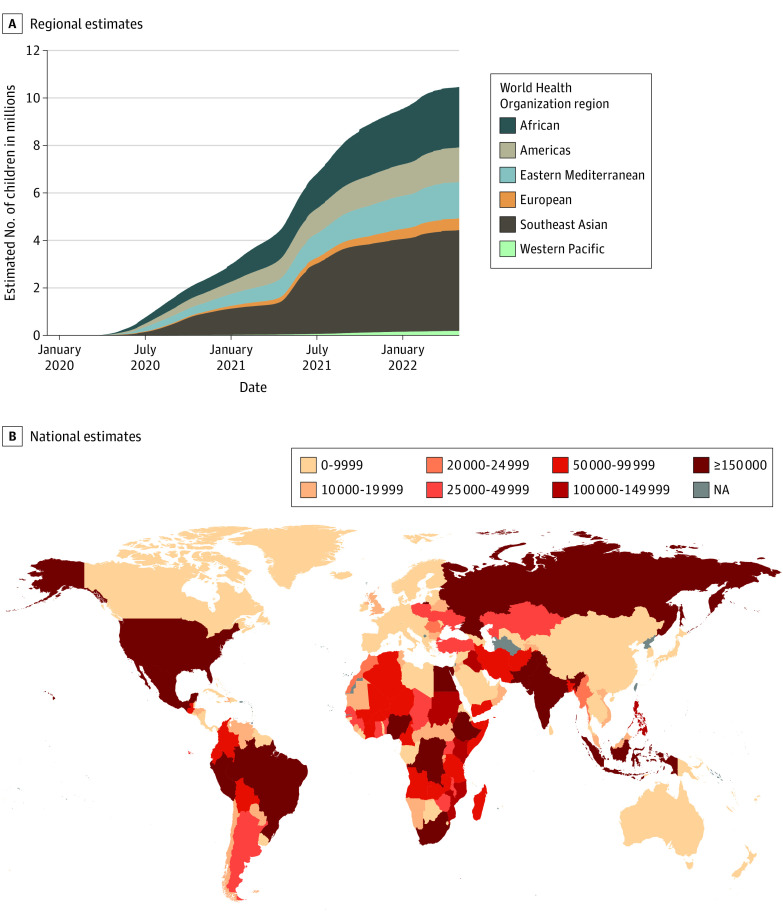
World Health Organization (WHO) Regional and National Estimates of Orphanhood and Primary and/or Secondary Caregiver Loss From January 1, 2020, Through May 1, 2022 We use excess death derived using WHO estimates through 2021 (end of reporting period). For January 1, 2022, to May 1, 2022, we extrapolated estimated excess deaths for WHO and the Institute for Health Metrics and Evaluation by adjusting Johns Hopkins University data (classification of countries by WHO region as previously reported^4^). NA indicates not applicable.

## Discussion

COVID-19–associated orphanhood and caregiver death has left an estimated 10.5 million children bereaved of their parents and caregivers. While billions of dollars are invested in preventing COVID-19–associated deaths, little is being done to care for children left behind. However, billions of dollars invested in supporting AIDS-orphaned children showcase successful solutions ready for replication.^4^ Only 2 countries, Peru and the US, have made national commitments to address COVID-19–associated orphanhood. At the 2nd Global COVID-19 Summit (May 12, 2022), President Biden emphasized the urgency of caring for the millions of children orphaned. Urgently needed pandemic responses can combine equitable vaccination with life-changing programs for bereaved children. An important limitation is that modeling estimates cannot measure actual numbers of children affected by caregiver death; future pandemic surveillance should include such children. Given the magnitude and lifelong consequences of orphanhood, integration into every national pandemic response plan of timely care for these children will help mitigate lasting adverse consequences. Evidence highlights 3 essential components: (1) prevent death of caregivers by accelerating vaccines, containment, and treatment; (2) prepare families to provide safe and nurturing alternative care; and (3) protect orphaned children through economic support, violence prevention, parenting support, and ensuring school access. Effective, caring action to protect children from immediate and long-term harms of COVID-19 is an investment in the future and a public health imperative.

## Notes

Supplement. eMethods.
