# Supplementary material for: Orphanhood and Caregiver Loss Among Children Based on New Global Excess COVID-19 Death Estimates
Source: JAMA Pediatr. 2022 Sep 6;176(11):1145–8. doi: 10.1001/jamapediatrics.2022.3157 (PMC9449868; doi:10.1001/jamapediatrics.2022.3157)
Supplement: Supplement. — eMethods. [file jamapediatr-e223157-s001.pdf]

Supplemental Online Content

Hillis S, N'konzi JN, Msemburi W, et al. Orphanhood and caregiver loss among children based on new global excess COVID-19 death estimates. *JAMA Pediatr*. Published online September 6, 2022. doi:10.1001/jamapediatrics.2022.3157

eMethods

This supplemental material has been provided by the authors to give readers additional information about their work.

© 2022 Hillis S et al. *JAMA Pediatrics.*

eMethods.

We adapt previously published methods[4] to estimate orphanhood and caregiver loss from excess death estimates made by The Economist[2] , The Institute of Health Metrics and Evaluation (IHME)[1] and the World Health Organization (WHO)[3]. Based on our study being a modeling study, we used the Guidelines for Accurate and Transparent Health Estimates Reporting. Excess deaths are defined as the number of deaths from all causes during a crisis above and beyond what we would have expected to see under normal conditions for the same time frame. At the time of writing, these are the only three global estimates of excess deaths and they enable us to account for underreporting in COVID-19 data that exists in standard COVID-19 death data. Despite not all caregivers dying from COVID in these data, children will have lost care during the pandemic period and so usual help and practices would have been disrupted.

We consider three different categories: orphanhood, primary caregiver loss and primary and/or secondary caregiver loss. We defined orphanhood using UNICEFs definition of the loss of one or more parents; primary caregiver death as death of parents or custodial coresident grandparents (providing care for children in the absence of parents); and secondary caregiver death as death of coresident grandparents or other older kin (providing care through involvement or resources). [4]

In Unwin et al.,[4] we use a logistic regression model to calculate orphanhood and caregiver loss ratios to deaths based on total fertility rate and a fixed effect for Western Europe. We fit this model to data calculated from 21 study countries (Argentina, Brazil, Colombia, England & Wales, France, Germany, Kenya, Malawi, Mexico, Nigeria, India, Iran (Islamic Republic of), Italy, Peru, the Philippines, Poland, Russian Federation, Spain, South Africa, United States of America, and Zimbabwe). In the current report, instead of using COVID-19 death data from Johns Hopkins University for all countries as in our most recent report,[4] we multiply our orphanhood and caregiver loss to death ratio by the maximum between excess deaths and COVID-19 deaths, which we describe as 'composite deaths,' for every country with

© 2022 Hillis S et al. *JAMA Pediatrics.*

data, from two time periods: January 1, 2020, through December 31, 2021 (end of reporting period from IHME[1] and WHO[3] datasets) and January 1, 2020, through May 1, 2022 for each of our three datasets. This is consistent with previous methodology.[4] WHO data for Kenya excess deaths are incomplete, so for the current analysis, we use methods described in our Lancet Child and Adolescent Health paper to estimate total COVID-19 deaths (by multiplying the COVID-19 death data from Johns Hopkins by 10 to account for the high prevalence of underreporting of deaths in Africa).[4]

We use a death adjustment factor for each country to update our data outside the time-period for the IHME and WHO data, calculated on December 31, 2021, when we have data for both excess and COVID-19 deaths. Specifically, we assume the ratio of excess deaths to COVID-19 deaths through Dec 31, 2021, was consistent with the ratio between January 1, 2022, and May 1, 2022, for WHO and IHME estimates. We, therefore, multiplied the new COVID-19 deaths by the ratio for the prior period to generate an estimate of country-specific excess deaths for this new period. If COVID-19 deaths were greater than excess deaths, we used the reported value of COVID-19 deaths. The Economist data are released weekly and available through May 1, 2022, so no data adjustment factor is needed. The Economist dataset includes 224 countries, IHME includes 190 countries, and estimates based on WHO methods include 181 countries, due to how the data are grouped.

We consider uncertainty in both the total fertility rate and excess deaths using bootstrapping, similar to methods previously described[4]. For all three data sets, we assume the total fertility rate is normally distributed with the standard deviation based on the lower and upper bounds. We do not allow the total fertility rate to vary for our 21 study countries and use the ratios generated for the study opposed to ones estimated from the logistic model. For the IHME and Economist data we also assume the number of excess deaths were normally distributed with the standard deviation based on the lower and upper bounds. However, for the WHO data we sample the excess deaths with replacement from their 1000 Markov Chain Monte Carlo draws. Consistent with previous methodology[4], we assume deaths were

© 2022 Hillis S et al. *JAMA Pediatrics.*

constant across all samples if COVID-19 deaths were greater then excess deaths. We use 5,000 samples for our bootstrap since at this number of samples, the error has begun to converge.

Differences exist between our estimates of orphanhood and caregiver loss due to the underlying excess death data from our three datasets. Methodological differences and data used to fit the models differ between the three datasets, which result in the three different estimates of excess deaths we use as an input in our model. We choose WHO estimates for our focus, as these are the most conservative.

© 2022 Hillis S et al. *JAMA Pediatrics.*
